# Cisplatin-induced ototoxicity in organotypic cochlear cultures occurs independent of gap junctional intercellular communication

**DOI:** 10.1038/s41419-020-2551-8

**Published:** 2020-05-11

**Authors:** Julia Abitbol, Rianne Beach, Kevin Barr, Jessica Esseltine, Brian Allman, Dale Laird

**Affiliations:** 1 Department of Anatomy and Cell Biology, Schulich School of Medicine and Dentistry, University of Western Ontario, London, ON N6A 5C1 Canada; 2 Division of BioMedical Sciences, Faculty of Medicine, Memorial University of Newfoundland, St. John’s, NL A1B 3V6 Canada

**Keywords:** Cell biology, Mechanisms of disease

## Abstract

Cisplatin is a very effective chemotherapeutic, but severe and permanent hearing loss remains a prevalent side effect. The processes underpinning cisplatin-induced ototoxicity are not well understood. Gap junction channels composed of connexin (Cx) subunits allow for the passage of small molecules and ions between contacting neighboring cells. These specialized channels have been postulated to enhance cisplatin-induced cell death by spreading “death signals” throughout the supporting cells of the organ of Corti. This study sought to investigate the role of Cx43 in cisplatin-induced ototoxicity using organotypic cochlear cultures from control and two Cx43-mutant mouse strains harboring either a moderate (Cx43^I130T/+^) or severe (Cx43^G60S/+^) reduction of Cx43 function. Cochlear cultures from Cx43-mutant mice with a severe reduction in Cx43-based gap junctional intercellular communication (GJIC) had an enhanced number of hair cells that were positive for cleaved caspase 3, a marker of active apoptosis, after cisplatin treatment. In cisplatin-treated organotypic cochlear cultures, there was a decrease in the co-localization of Cx26 and Cx30 compared with untreated cultures, suggesting that cisplatin causes reorganization of connexin composition in supporting cells. Both Cx26 and Cx30 protein expression as well as GJIC were decreased in organotypic cochlear cultures treated with the gap-junction blocker carbenoxolone. When cisplatin and carbenoxolone were co-administered, there were no differences in hair cell loss compared with cisplatin treatment alone. Using cisplatin-treated control and Cx43-ablated organ of Corti derived HEI-OC1 mouse cells, we found that greatly reducing GJIC led to preferential induction of an ER stress pathway. Taken together, this study strongly suggests that inhibition of GJIC in organ of Corti cells does not lead to differential susceptibility to cisplatin-induced ototoxicity. Although cisplatin causes the same degree of cell death in gap junction competent and incompetent cochlear cells, the engagement of the mitochondrial dysregulation and ER stress differs.

## Introduction

Connexins, the protein subunits that form gap junction channels, allow for the passage of members of the metabolome up to 1 kDa in size, establishing a form of cellular signaling called gap junctional intercellular communication (GJIC)^1,2^. Mounting evidence suggests that connexin hemichannels where cochlear cells communicate with the extracellular milieu also serve important functions^3^. Gap junctions are essential to maintain proper tissue homeostasis as evidenced by nearly 30 distinct diseases that are associated with connexin gene mutations, with hearing loss being the most frequently occurring impairment^4^. Clinically, mutations and/or deletions in *GJB2* (encoding Cx26) and/or *GJB6* (encoding Cx30) are responsible for nearly 50% of congenitally acquired hearing loss with ~135 different mutations in *GJB2* causing hearing loss^4,5^.

Spontaneous activity in the cochlea depends upon ATP and calcium release, suggesting a critical role for connexins in cochlear development^3,6^. The necessity of connexins in the development of the organ of Corti (i.e., the sensory epithelium in the cochlea) is revealed from the use of Cx26 conditional knockout mice where hair cell loss and underdevelopment of the organ of Corti leads to hearing loss^7–9^. Complementary studies using tamoxifen-induced Cx26 knock-down mice revealed that Cx26 was a key regulator in early cochlear development. Indeed, knocking down Cx26 in early postnatal stages resulted in severe hearing loss, malformation of the cochlea, and defects in supporting cells^10–13^.

The localization and expression pattern of Cx43 in the cochlea remains controversial, but Cx43 has been reported to be expressed at distinct developmental time points in the organ of Corti^14–16^, spiral limbus^17^, spiral ganglion neurons^18–20^, cochlear lateral wall^21^, cochlear nerve, and auditory brainstem tract^22^. In keeping with a key role for Cx43 in hearing, we previously showed that the severe loss of Cx43 function led to hearing loss^23^, suggesting that Cx43 plays an important role in the development and/or function of the auditory pathway. That said, it remains unclear if dysregulated Cx43 status during development influences the susceptibility of cochlear cells to drug-induced cell death and hearing loss.

Cisplatin (*cis*-diamminedichloroplatinumII) is one of the most effective and commonly used chemotherapy drugs to treat solid malignant tumors in children and adults, often in combination with surgery and/or radiation treatment^24–26^. Although commonly used, it causes hearing loss in ~75–100% of patients. In most cases, cisplatin-induced hearing loss is permanent, and progressive with continued cisplatin treatment. Most cisplatin-induced hearing loss occurs within the basal (high-frequency) region^27^. Notably, ~60% of children who are treated with cisplatin will develop significant hearing loss ranging from mild-to-severe, which can hinder their cognitive and speech skills throughout development^28,29^. Within the cochlea specifically, animal models have shown that cisplatin causes damage to the stria vascularis, spiral ganglion neurons, and hair cells^30–32^. Supporting cells of the organ of Corti have some of the largest and most active gap junctions found in the human body^33^. It has been proposed in other systems that gap junction channels facilitate the spread of toxic death signals to adjoining cells amplifying cell death in a process called the “bystander effect”^34^. Specifically, Cx43 has been reported to exacerbate cisplatin-induced cell death in a cochlear-relevant cell line^35^. Depending on the cells being studied, the bystander effect may also act as a “Good Samaritan” by diluting the effect of toxins^34^. Within the organ of Corti, bystander-mediated toxin dilution could protect cells from increased damage. To address the cellular mechanisms of cisplatin-induced ototoxicity, we utilized mouse organ of Corti cultures that retain the three-dimensional aspects and architecture of this sensory organ in addition to a well-used mouse immortalized cochlear cell line, HEI-OC1, which resembles cochlear progenitor cells^36^.

The aims of this study were threefold. We first used organotypic cochlear cultures from two Cx43 mutant mice, which harbor either a moderate (Cx43^I130T/+^) or severe (Cx43^G60S/+^) loss of Cx43 function to determine the impact of reduced GJIC on cisplatin-induced ototoxicity. Second, we co-administered a gap junction blocker with cisplatin to determine whether blocking all cochlear gap junctions alters susceptibility to cisplatin-induced ototoxicity. Third, we used control and Cx43 knockout HEI-OC1 cochlear-derived cells to evaluate the cellular mechanisms underpinning cisplatin-induced ototoxicity.

## Materials and methods

### Mice

Mice heterozygous for the Cx43 I130T mutation (provided by Glenn Fishman, New York University School of Medicine, New York, NT, USA) were previously described^37^ and backcrossed as we previously noted^23^. Mice heterozygous for the Cx43 G60S mutation (provided by Janet Rossant, The Hospital for Sick Children, Toronto, ON, Canada) were previously described^38^ and backcrossed as we previously noted^23^. Both mutant mice were compared with their respective wild-type (WT) littermates for all experiments, and both male and female neonatal pups were used. WT C3H mice were used for all experiments where carbenoxolone was employed as a gap junction blocker. Mice were maintained at the University of Western Ontario in a 12-h-light and dark cycle. All animal studies were reviewed and authorized by the Animal Care Committee at the University of Western Ontario. Mice were randomized between different treatment groups.

### Organotypic cochlear cultures

Organotypic cochlear cultures were generated from the cochleae of postnatal day 0–3 (P0–P3) pups of both sexes, combined, and maintained in glass bottom dishes, as was previously described^23^. Briefly, mouse pups were sacrificed by decapitation, sterilized in 80% ethanol for 10 min, and dissected in ice-cold Leibovitz-L15 media (Invitrogen, catalog# 11415-064). The epithelium of the organ of Corti was removed, and the explants were plated in a glass bottom dish coated with Cell Tak (Corning; catalog# CB40240) to enhance adhesion to the substrate. Cultures were submerged in 200 μL of Dulbecco’s Modified Eagle’s Medium (DMEM)/F12 medium containing 5% fetal bovine serum (FBS) (Invitrogen), and 100 μg/mL ampicillin. Cultures were incubated at 37 °C and 5% CO_2_ overnight after dissection to ensure proper adhesion. Subsequently, cultures were treated with either regular media or 20 μM cisplatin (within the therapeutic range of cisplatin administration in the clinical setting)^39,40^, 100 μM carbenoxolone (CBX) (a concentration that has been shown to effectively block GJIC in the inner ear)^41,42^, or a combination of 20 μM cisplatin + 100 μM CBX for a total of 48 h prior to fixing with 4% paraformaldehyde (PFA). Treatment groups were randomized between different cultures. Cisplatin (Sigma, catalog# PHR1624) was prepared fresh in a saline solution according to the manufacturer specifications to a final concentration of 10 mM. This stock cisplatin solution was subsequently diluted in DMEM media to the desired concentrations and was freshly prepared for each experiment. Media for all organotypic cochlear cultures was aspirated and replaced every 24 h for all respective treatments.

### Immunofluorescence labeling

Fixed cell cultures were blocked and permeabilized in either a 3% bovine serum albumin (BSA) + 0.1% Triton X-100 (HEI-OC1 cells) or 3% BSA + 0.2% Triton X-100 (organotypic cochlear cultures) solution for 1 h. Subsequently, cell cultures were incubated at 4 °C overnight with primary antibodies, with the exception of antibodies to cleaved caspase3 which was incubated for 2 h at room temperature. These antibodies included; rabbit anti-Cx30 (1:400, Thermo-Fisher, catalog# 71-2200), mouse anti-Cx26 (1:400, Thermo-Fisher, catalog# 33-5800), rabbit anti-Cx43 (1:500, Sigma, catalog# C6219), rabbit anti-MyosinVI (1:200, Proteus Biosciences, catalog# 25-6791), rabbit anti-cleaved caspase 3 (1:1000, Sigma, catalog# C9598), mouse anti-protein disulfide isomerase (PDI) (1:500, Assay Designs, catalog# SPA-891), and anti-BiP (1:1000, Sigma-Aldrich, catalog# G8918). Cell cultures were washed and incubated with fluorescent-conjugated secondary antibodies for 1 h followed by a nuclear Hoechst 33342 stain. Cultures were additionally stained with phalloidin (1:400, Invitrogen, catalog# A12379). HEI-OC1 cells were mounted on a glass slide with airvol. A Zeiss LSM800 confocal microscope was used to acquire Z-stacks and high-resolution Airyscan images. For all quantifications performed on organotypic cochlear cultures (i.e., hair cell counts and cleaved caspase 3-positive hair cell counts), the experimenter was blinded to treatment group, genotype, and cochlear region when counting. *N* values for each experiment are described in figure legends.

### Co-localization and particle analysis

Organotypic cochlear cultures from Cx43^G60S/+^ mutant mice and their WT littermates were treated with regular media or cisplatin prior to immunolabeling for Cx26 and Cx30, and subsequent co-localization and particle analysis. A Zeiss LSM800 confocal microscope was used for calculating Pearson’s correlation coefficient with a co-localization plug-in. Controls of single-labeled cultures (i.e., only Cx26 or only Cx30 primary antibodies) were used to determine thresholds of intensities for each single channel and to set up bin crosshairs in scatterplots needed for analysis. Individual bins were set for all three cochlear turn regions (apical, middle, and basal) with the single-labeled controls. Once threshold values were established, Pearson’s correlation coefficient was used to measure co-localization of Cx26 and Cx30. The same images used for Pearson’s correlation analysis were used in ImageJ with the “analyze particles” function to quantify the number of gap junction plaques, their size, as well as their average pixel intensities under the same experimental parameters. All Airyscan images used for both co-localization and particle analysis were obtained from Kolliker’s area of the cultures using the 63x × 1.4 NA oil immersion lens, where imaging parameters including laser intensity and number of stacks were maintained for all groups.

### Fluorescence recovery after photobleaching (FRAP)

Control and drug-treated organotypic cochlear cultures were incubated for 48 h. Cultures were then washed with phosphate-buffered saline (PBS) and incubated for 5 min at room temperature in 2 μM calcein-AM dye (Invitrogen, catalog# 3100-MP) in DMEM/F12 medium. Cultures were next washed twice with PBS, and fresh DMEM/F12 medium was added. Dye loaded cultures were placed in a live cell incubation chamber (37 °C, 5% CO_2_) under a Zeiss LSM800 confocal microscope. To assess the level of gap junction function, both the outer sulcus and inner sulcus regions of organotypic cultures were selected for FRAP analysis. One outer sulcus cell that was adjacent to at least three other cells was photobleached, and the recovery of fluorescence into the photobleached cell was examined every 10 s for eight minutes. Defined regions (288.5 µm^2^ in diameter) of the tightly packed cells of the inner sulcus region were photobleached, and fluorescence recovery to the photobleached region was determined as described above. The intensities of fluorescence were measured in ImageJ, and the percent recovery was calculated as; (F_x_ − F_P_/F_I_) × 100, F_x_ = fluorescence at each time point, F_P_ = fluorescence after photobleaching, and F_I_ = initial fluorescence before photobleaching. Area under the curve of a linear regression analysis was calculated using GraphPad Prism 6.

### Cell culture and reagents

House Ear Institute–Organ of Corti 1 (HEI-OC1) mouse cells were kindly provided by Dr. Kalinec (House Ear Institute, Los Angeles, CA). HEI-OC1 cells were grown in high glucose DMEM supplemented with 10% FBS and 2 mM L glutamine, and incubated in permissive conditions (33 °C, 10% CO_2_). Once HEI-OC1 cells reached 80% confluency, they were treated with various doses of cisplatin (Sigma-Aldrich, catalog# PHR1624) or saline as a vehicle control (referred to as untreated) for 24 or 48 h. Positive controls for the induction of apoptosis in HEI-OC1 cells were treated with 1 μM staurosporine (Sigma, catalog# S6942) for 2 h. Positive controls for the induction of ER stress in HEI-OC1 cells were treated with 2 μM thapsigargin (Sigma, catalog# T9033) for 4 h.

### CRISPR-Cas9 gene ablation

HEI-OC1 cells were subjected to CRISPR-Cas9 ablation of the *Gja1* gene, as we have previously described^43^. Two gRNAs were engineered using the Sanger Institute CRISPR finder (http://www.sanger.ac.uk/htgt/wge/) (mouse *Gja1*: Sanger sgRNA ID: 324658622 (5′-CGCTGTAACACTCAACAACC-3′) and Sanger sgRNA ID: 324658605 (5′-AAGCCTACTCCACGGCCGGA-3′)). HEI-OC1 cells expressing the reporter green fluorescent protein were sorted using fluorescence-activated cell sorting (FACS), and at least two Cx43-ablated clones were confirmed and used for each experiment as we previously described^43^.

### WST-1 assay

Cell viability was analyzed using the WST-1 assay (Sigma, catalog# 5015944001). HEI-OC1 cells were plated at a density of 1 × 10^4^ cells (low density) or 3 × 10^4^ cells (high density) in each well of a 96-well dish and incubated overnight. HEI-OC1 cells were treated with the intended concentration of cisplatin in triplicate for 24 or 48 h. HEI-OC1 cells were then treated with 1:10 dilution of WST-1 in fresh media for 2 h. Wells with no cells or media only were used as a negative control. The optical density of each sample was measured using a spectrophotometer reader at 450 nm and a reference wavelength at 630 nm. Cell viability was determined by subtracting the reference absorbance and the optical density of the negative control from each sample. Cell viability was then measured as a percentage of the cell viability of cells treated with saline, denoted as 100%.

### Immunoblotting

Control or drug-treated HEI-OC1 cells were washed with PBS and lysed using 1× IP lysis buffer ((2× IP lysis buffer (2 % Triton X-100, 330 mM NaCl, 20 mM Tris, 2 mM EDTA, 2 mM EGTA, 1% NP-40, pH 7.4)), containing 1 tablet complete mini protease inhibitor cocktail (Sigma-Aldrich), 1:100 sodium fluoride (Millipore Sigma, catalog# SX0550-1) and 1:100 sodium orthovanadate (Sigma, catalog# S6508) phosphatase inhibitors in PBS. Once protein concentrations were measured, equal amounts of protein were resolved using SDS-PAGE (10% or 12% polyacrylamide gel), and were transferred to a nitrocellulose membrane using an iBlot transfer system. Membranes were probed overnight with primary antibodies in 3% BSA dissolved in PBS Tween (0.05% Tween 20), unless indicated otherwise. Primary antibodies included: mouse anti-GAPDH (1:5000, EMD Millipore, catalog# MAB374), rabbit anti-Bax (dissolved in Tris-buffered saline (TBS) Tween, 1:1000, Cell Signaling, catalog# 2772), and rabbit anti-BiP (1:1000, Sigma-Aldrich, catalog# G8918). Membranes were then washed with PBS or TBS Tween and incubated with Alexa Fluor 680-conjugated anti-rabbit-IgG secondary antibody (1:5000, Life Technologies, catalog# A21057) or IRdye800-conjugated anti-mouse-IgG antibody (1:5000, Rockland, catalog# 611-132-002) for 1 h. Protein signals were then visualized using an Odyssey infrared imaging system (LiCor), and the intensity of the bands were quantified using Odyssey software. Protein expression was normalized to the loading control GAPDH.

### Quantitative reverse transcriptase polymerase chain reaction

Quantitative reverse transcriptase polymerase chain reaction (qRT-PCR) was performed as we recently described^43^. The following primers were used: 18s rRNA, the house keeping gene (forward, 5′-GTAACCCGTTGAACCCCATT; reverse, 5′-CCATCCAATCGGTAGTAGCG), manganese superoxide dismutase (MnSOD) (forward, 5′-GGCCAAGGGAGATGTTACAA; reverse, 5′-CCTTGGACTCCCACAGACAT), glutathione peroxidase (GPx1) (forward, 5′-GTCCACCGTGTATGCCTTCT; reverse 5′-CCTCAGAGAGACGCGACATT), catalase (forward, 5′-GGAGGCGGGAACCCAATAG; reverse, 5′-GTGTGCCATCTCGTCAGTGAA, Bcl-2 (forward, 5′-CCTGTGGATGACTGAGTACC; reverse, 5′-GAGACAGCCAGGAGAAATCA), Bax (forward, 5′-GTTTCATCCAGGATCGAGCA; reverse, 5′-CATCTTCTTCCAGATGGTGA). WT was set as a control, and mRNA levels were normalized to 18s rRNA and measured using the ΔΔCT method.

### Statistical analysis

Two-way analysis of variance (ANOVA) followed by Tukey’s post hoc tests were used for hair cell counts, cell viability, cleaved caspase 3 staining, mRNA and protein analysis, and gap junction particle analysis at each individual cochlear region (i.e., apical, middle, and basal). A one-way ANOVA followed by Tukey’s post hoc tests were performed for analyzing cleaved caspase 3 staining of organotypic cochlear cultures, and Pearson’s correlation coefficient of each cochlear region. A regression line analysis was performed for percent of dye recovery, which was used to generate the area under the curve. Additional one-way ANOVAs followed by Tukey’s post hoc tests were performed for HEI-OC1 cell experiments, and are denoted in figure legends. Sample size estimates, estimates of variation, as well as statistical tests used were chosen based on previous studies and specific sample sizes used are designated in figure legends. For all cell count analyses, the experimenter was blinded to treatment groups. All statistical analysis was performed using GraphPad Prism 6.

## Results

### Cx26 and Cx30 are abundantly expressed in organotypic cochlear cultures while Cx43 is detected at low levels

**Fig. 1:**
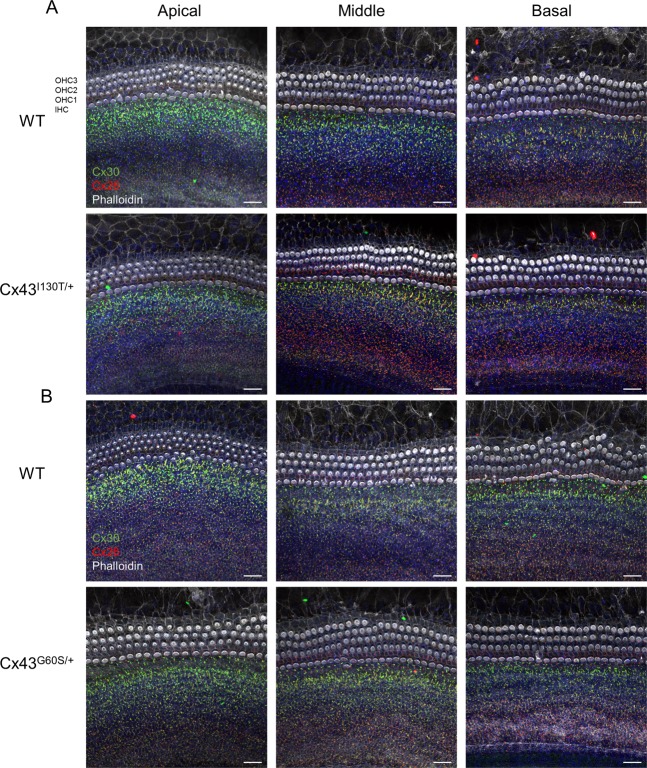
Cx26 and Cx30 are highly expressed in supporting cells of organotypic cochlear cultures. **a** Representative confocal images of Cx26 and Cx30 distribution in organotypic cochlear cultures from WT and Cx43^I130T/+^ mutant mice denoting the apical (low-), middle (mid-), and basal (high-frequency) regions. **b** Representative confocal images of Cx26 and Cx30 distribution in organotypic cochlear cultures of WT and Cx43^G60S/+^ mutant mice. OHC1 outer hair cell row 1, OHC2 outer hair cell row 2, OHC3 outer hair cell row 3, IHC inner hair cell. Cx30 is denoted in green, Cx26 is in red, phalloidin labeling hair cell stereocilia is in white, and Hoechst nuclear stain is in blue. Scale bars = 20 μm.

To assess the connexin content of organotypic cochlear cultures from Cx43 mutant and WT littermate mice, cultures were immunolabeled for Cx26 and Cx30. High levels of Cx26 and Cx30 protein were found in Kolliker’s organ supporting cells of the cochlea (Fig. 1a, b). Of note, there were no detectable changes in the distribution or levels of Cx26 or Cx30 in either Cx43-mutant mice compared with their respective WT littermates (Fig. 1a, b). In all cases, both Cx26 and especially Cx30 appeared to be more predominantly found in the apical (low-frequency) regions compared with the basal (high-frequency) regions, which was most apparent in the Cx43^G60S/+^ mutant mice and their WT littermates (Fig. 1b).

**Fig. 2:**
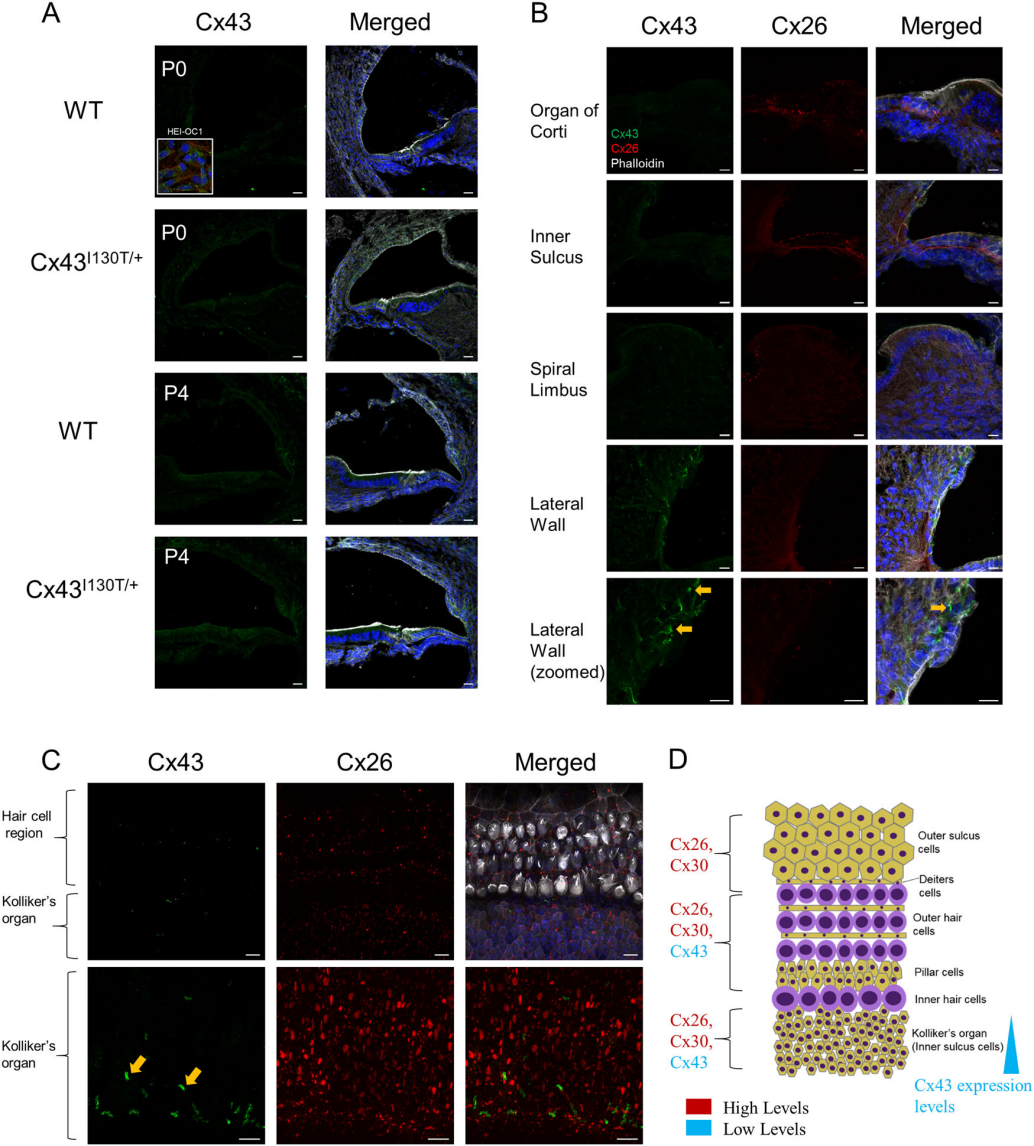
Low but detectable amounts of Cx43 are expressed in organotypic cochlear cultures. **a** 3D reconstruction of consecutively stacked confocal images of cochleae obtained from Cx43^I130T/+^ mutant mice and their WT littermates (P0 and P4) apical regions. Insert = HEI-OC1 cells. **b** Higher-magnification images of specific cochlear regions of WT P4 cochleae. **c** Stacked confocal images of organotypic cochlear cultures from WT mice of apical regions. **d** Schematic diagram representing the localization and relative expression levels of cochlear connexins in organotypic cochlear cultures. Cx43 is denoted in green, Cx26 is in red, phalloidin is in white, and Hoechst nuclear stain is in blue. Orange arrowheads indicate areas of gap junction plaques. Scale bars = 10 μm.

To examine the localization of Cx43 in the cochlea, Cx43 immunofluorescence labeling was performed on cross-sections from young WT and mutant mice (P0 and P4) as well as organotypic cochlear cultures from WT mice. Low magnification confocal images of P0 and P4 cochleae revealed no Cx43 gap junctions in the organ of Corti (Fig. 2a). Higher-magnification confocal images also revealed no evidence for Cx43 in the organ of Corti, inner sulcus, or spiral limbus regions (Fig. 2b). However, detectable Cx43 expression was found in the cochlear lateral wall region and in supporting cells of Kolliker’s organ within organotypic cochlear cultures with higher expression in the inferior region of Kolliker’s organ (Fig. 2b, c). A diagrammatic representation of the relative distribution patterns of Cx26, Cx30, and Cx43 in organotypic cochlear cultures is outlined (Fig. 2d).

### Cisplatin causes hair cell death in Cx43-mutant mice and their WT littermates

**Fig. 3:**
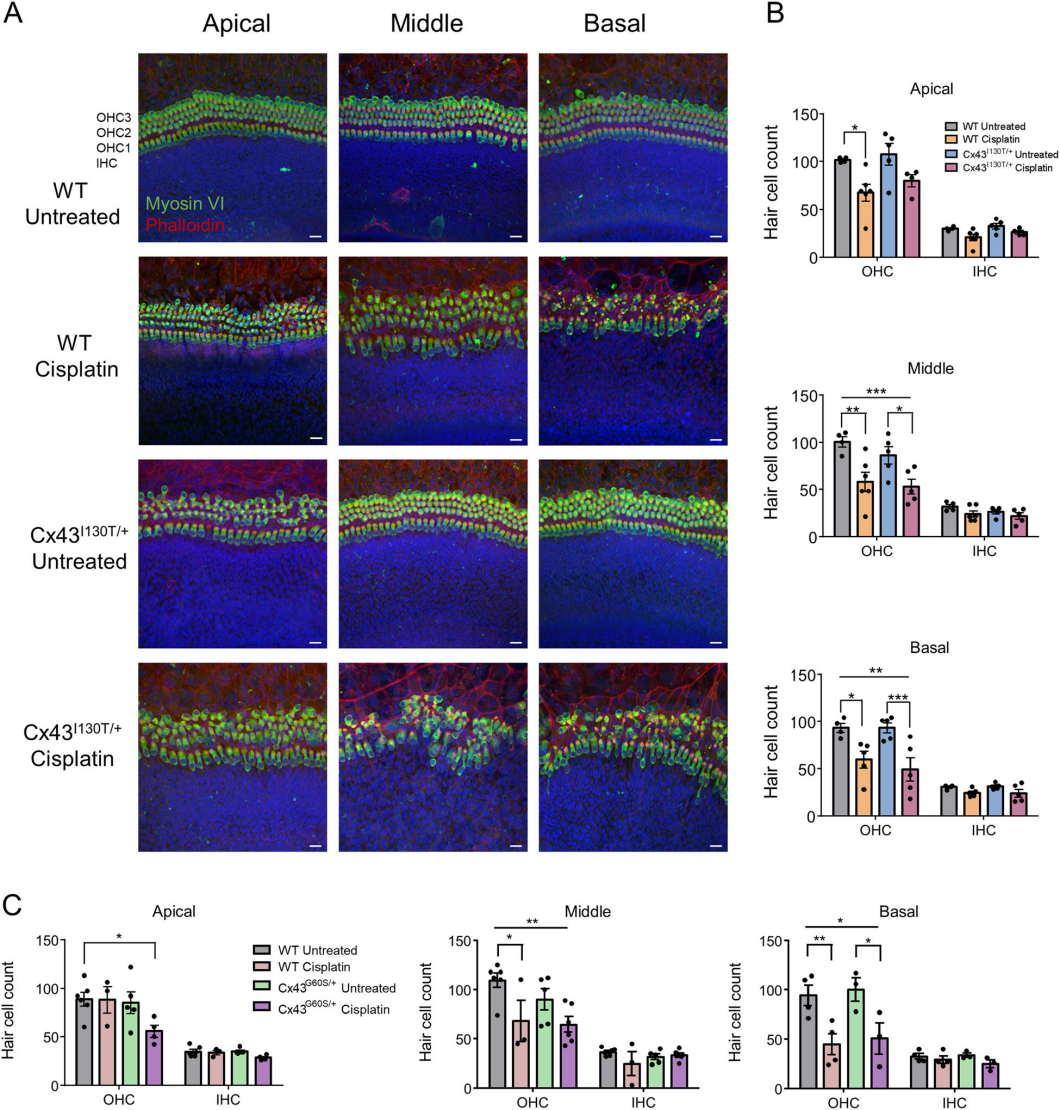
Cisplatin causes hair cell loss in organotypic cochlear cultures from WT and Cx43^I130T/+^ mutant mice. **a** Representative confocal images of organotypic cochlear cultures from WT and Cx43^I130T/+^ mutant littermate mice either untreated or treated with 20 μM cisplatin for 48 h. MyosinVI was immunolabeled to denote the hair cell body (green), phalloidin staining was used to demarcate actin (red), and Hoechst staining was used to locate the position of the nuclei (blue). **b** Quantitative analysis of hair cell counts in apical, middle, and basal regions of Cx43^I130T/+^ and WT littermate mouse cultures, either untreated or treated with 20 μM cisplatin. N values; WT untreated = 4, WT cisplatin = 5–6, Cx43^G60S/+^ untreated = 5, Cx43^G60S/+^ cisplatin = 4–5. **c** Quantitative analysis of hair cell counts in apical, middle, and basal regions of cultures for Cx43^G60S/+^ mutant mice and WT littermates. N values; WT untreated = 4–6, WT cisplatin = 3–4, Cx43^G60S/+^ untreated = 3–5, Cx43^G60S/+^ cisplatin = 3–6. OHC outer hair cell, IHC inner hair cell. Means represent mean ± standard error. Scale bars = 20 μm. Two-way ANOVAs were performed for each cochlear region with a subsequent Tukey’s post hoc test. *p < 0.05, **p < 0.01, ***p < 0.001.

Since we know that low levels of Cx43 are present in distinct regions of the organ of Corti and Cx43 is important in the development of the organ of Corti, we wanted to determine if organotypic cultures from Cx43 mutant mice have differential susceptibility to cisplatin-induced hair cell loss. First, organotypic cochlear cultures from control and Cx43^I130T/+^ mutant mice were treated with 20 µM cisplatin for 48 h, and hair cells were immunolabeled for the hair cell body marker, MyosinVI, and phalloidin to visualize the hair cell stereocilia. There was no evidence of hair cell loss in untreated organotypic cochlear cultures from Cx43^I130T/+^ mutant mice (Fig. 3a). However, there was a significant decrease in the number of outer hair cells in all cochlear regions in cisplatin-treated cultures (Fig. 3b). Notably, there were no differences between WT and Cx43^I130T/+^ mutant hair cell loss after cisplatin treatment (Fig. 3b). To further examine whether a severe loss of Cx43 function led to differential hair cell death, organotypic cultures from control and Cx43^G60S/+^ mutant mice were treated with cisplatin. Cisplatin treatment caused a significant loss of outer hair cells in the middle and basal regions, however, there were no differences observed between WT cisplatin-treated and Cx43^G60S/+^ mutant cisplatin-treated organotypic cultures (Fig. 3c).

### Cisplatin induces regional changes in apoptosis in organotypic cultures from Cx43^G60S/+^ mutant mice

**Fig. 4:**
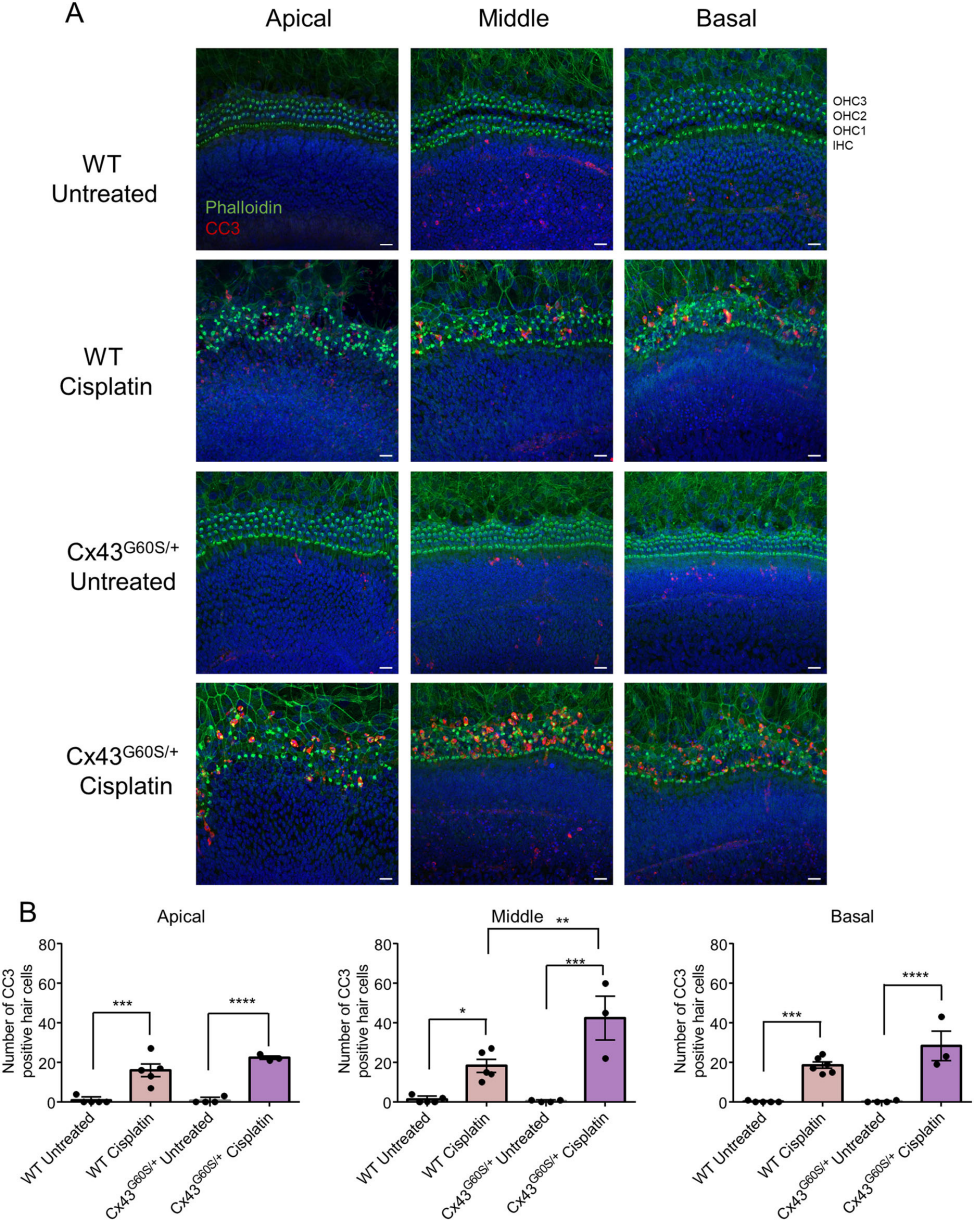
CC3 is preferentially upregulated in cochlear cultures obtained from Cx43^G60S/+^ mutant mice. **a** Representative confocal images of the apical, middle, and basal regions of organotypic cochlear cultures from WT and Cx43^G60S/+^ mice labeled for CC3 after being untreated or treated with 20 μM cisplatin. CC3 is denoted in red, phalloidin in green, and nuclei in blue. **b** CC3-positive hair cells were quantified as hair cells labeled positive for CC3 and phalloidin. N values; WT untreated = 5, WT cisplatin = 5–6, Cx43^G60S/+^ untreated = 4, Cx43^G60S/+^ cisplatin = 3. Means represent mean ± standard error. Scale bars = 20 μm. One-way ANOVA’s were performed for each cochlear region and subsequently a post hoc Tukey’s test was performed. *p < 0.05, **p < 0.01, ***p < 0.001, ****p < 0.0001.

Cisplatin most commonly causes damage to the inner ear through apoptosis. To examine whether reduced Cx43 function influences the level of apoptosis, we examined immunofluorescent staining of the late apoptosis marker, cleaved caspase 3 (CC3). In untreated cultures, no CC3-positive hair cells were found, however, after cisplatin treatment CC3-positive hair cells were readily identified indicative of apoptosis (Supplementary Fig. S1a, b). In all regions, there was a significant increase in the number of CC3-positive hair cells in cisplatin-treated WT cultures (Supplementary Fig. S1a, b). However, there were no differences in the number of CC3-positive hair cells between WT and Cx43^I130T/+^ mutant mice (Supplementary Fig. S1a, b). Similarly, the number of CC3-positive hair cells were significantly increased after cisplatin treatment in WT and Cx43^G60S/+^ mutant mice (Fig. 4a, b). Intriguingly, cisplatin-treated cultures from Cx43^G60S/+^ mutant mice had a significant increase in CC3-positive hair cells in the middle turn compared to cisplatin-treated WT littermates (Fig. 4a, b).

### Cx43 KO HEI-OC1 cells have similar sensitivity to cisplatin as WT cells

**Fig. 5:**
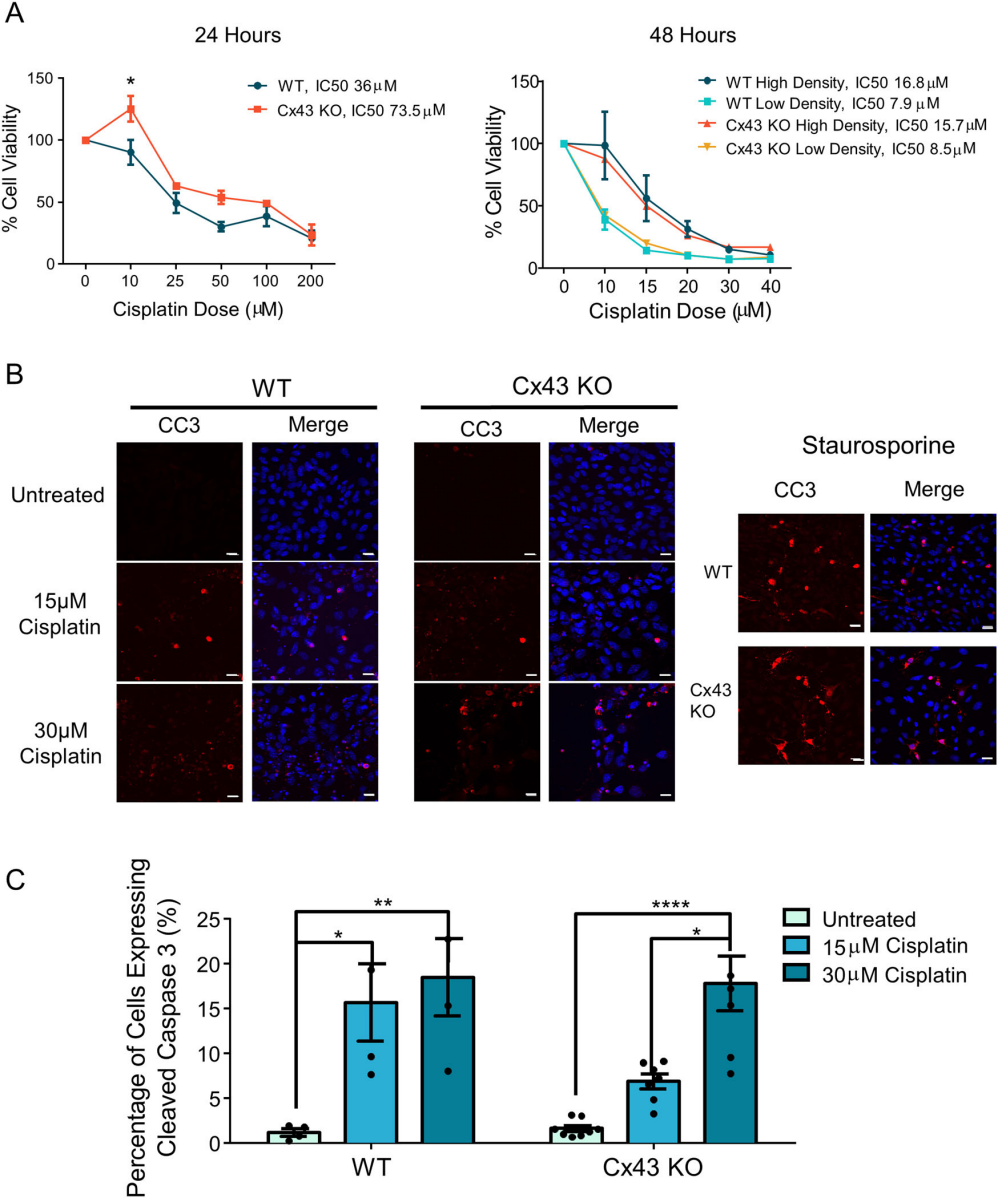
WT and Cx43-ablated HEI-OC1 cells both have reduced cell viability after cisplatin treatment. **a** Cell viability of HEI-OC1 cells at 24 and 48 h after cisplatin treatment. IC50s were calculated as the dose required to kill 50% of cells. **b** Representative confocal images of CC3 staining in control and cisplatin-treated cells. CC3 is denoted in red, and nuclei are in blue. Staurosporine-treated cells were used as a positive control. **c** Quantification of the percentage of CC3-positive cells showed a significant increase in the percentage of CC3-positive cells after cisplatin treatment. Bars represent mean ± standard error from four independent experiments comprised of two different Cx43 KO HEI-OC1 clones. *N* values; WT: *N* = 4, Cx43 KO: *N* = 7. Scale bars = 10 μm. Two-way ANOVA with Tukey’s post hoc test, *p < 0.05, **p < 0.01, ****p < 0.0001.

Dose-controlled cisplatin treatments for 24 h and 48 h revealed that both WT and Cx43 KO cells had greatly reduced cell viability (Fig. 5a). After 24 h of low dose (10 μM) cisplatin treatment, Cx43 KO cells were unaffected as compared with WT cells, however, this difference did not persist at higher cisplatin doses or at the 48-h time point (Fig. 5a). To assess whether the extent of gap junction formation may play a role in cisplatin-induced cell death, cells were plated at low density to minimize gap junction assembly. While the IC50 was reduced by 50% when cells were plated at low density, there was no difference between WT and Cx43 KO cells (Fig. 5a). The number of CC3-positive cells was significantly increased at both 15 μM and 30 μM cisplatin compared with untreated cells (Fig. 5b, c). However, no differences in the number of CC3-positive cells were observed between WT and Cx43 KO cells (Fig. 5c).

### Reduced Cx26 and Cx30 co-localization after cisplatin treatment of organotypic cultures

**Fig. 6:**
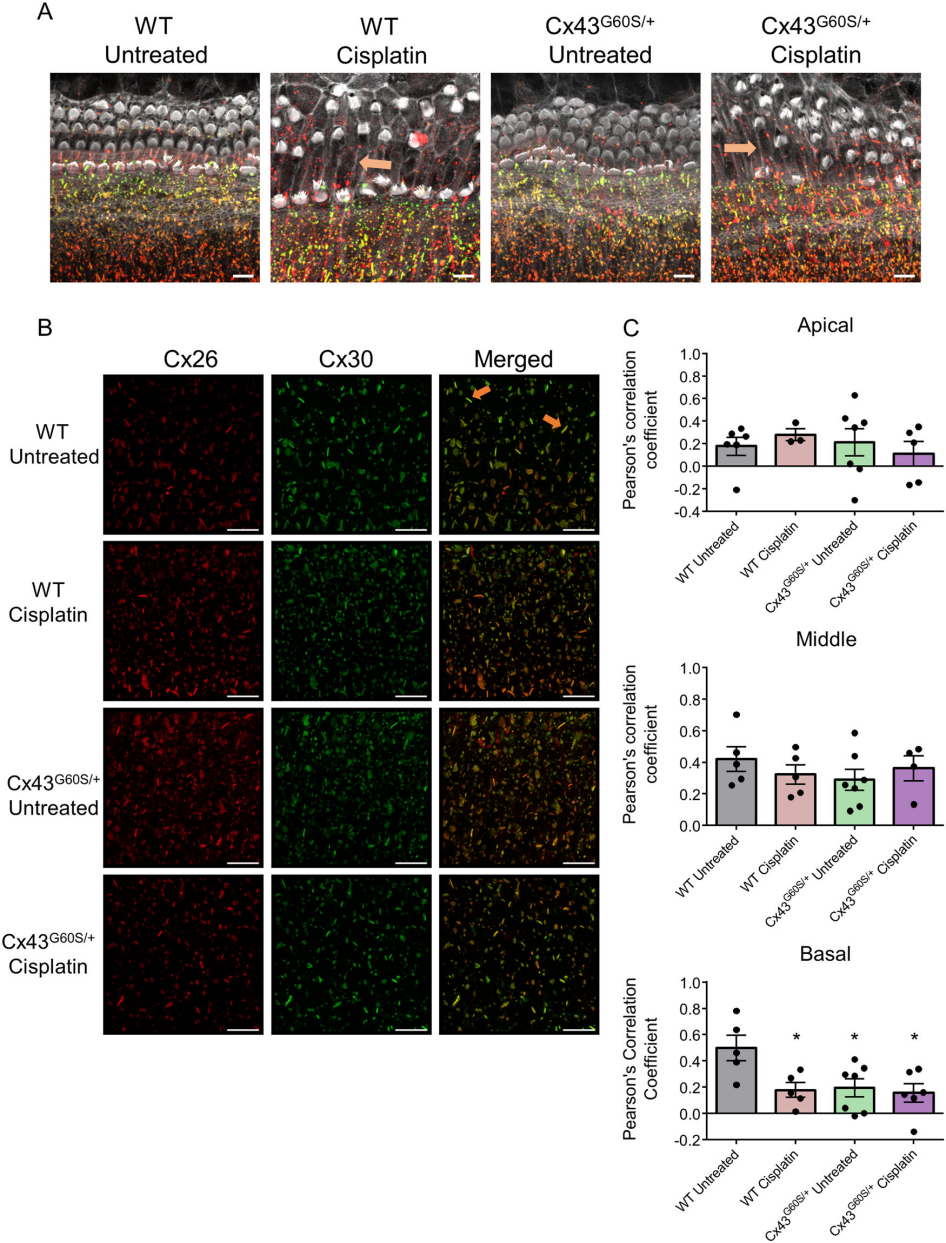
Cisplatin treatment alters the spatial location of hair cells, causes supporting cell expansion, and induces the reorganization of Cx26 and Cx30 gap junctions. **a** Representative confocal images of the basal region of organotypic cochlear cultures obtained from untreated and cisplatin-treated Cx43^G60S/+^ mutant mice and WT littermates. Orange arrows denote areas of pillar cell expansion. **b** High-magnification Airyscan images were acquired of the basal area from the inner sulcus region. Cx26 is denoted in red and Cx30 is in green. **c** Pearson’s correlation coefficient was quantified in apical, middle, and basal regions of organotypic cultures to measure Cx26 and Cx30 co-localization. *N* values; WT untreated = 5–6, WT cisplatin = 3–5, Cx43^G60S/+^ untreated = 6–7, Cx43^G60S/+^ cisplatin = 4–6. Means represent mean ± standard error. Scale bars = 10 μm. One-way ANOVA’s were performed for each cochlear region and subsequently a post hoc Tukey’s test was performed. *p < 0.05.

To assess whether cisplatin alters cochlear connexin distribution, double-immunolabeling for Cx26 and Cx30 was performed on control and cisplatin-treated cochlear cultures from WT and Cx43^G60S/+^ mutant mice. Representative confocal images of the basal region revealed altered hair cell morphology, hair cell loss, and supporting cell expansion, particularly in the pillar supporting cells (arrowheads), after cisplatin treatment (Fig. 6a). High-resolution Airyscan confocal images from the Kolliker’s organ were acquired and Pearson’s correlation coefficient was performed to assess the spatial localization of both Cx26 and Cx30. Cx26 and Cx30 were co-localized in untreated cultures within the basal region with a correlation coefficient of approximately 0.5, suggesting that 50% of Cx26 and Cx30 were co-localized (Fig. 6b, c). After cisplatin treatment, Cx26 and Cx30 were predominantly found separated into their own distinct gap junction plaques as co-localization was significantly decreased (Fig. 6b, c). Interestingly, both untreated and cisplatin-treated cultures from Cx43^G60S/+^ mutant mice had significantly decreased Cx26 and Cx30 co-localization compared with WT untreated cultures (Fig. 6b, c). Particle analysis was performed to determine the average intensity, number of gap junction plaques, and the size of gap junction plaques in untreated and cisplatin-treated cultures. No differences were found in any of the parameters tested in untreated and cisplatin-treated cultures (Supplementary Fig. S2a–c).

### Carbenoxolone decreases Cx26 and Cx30 levels and GJIC in WT organotypic cochlear cultures

**Fig. 7:**
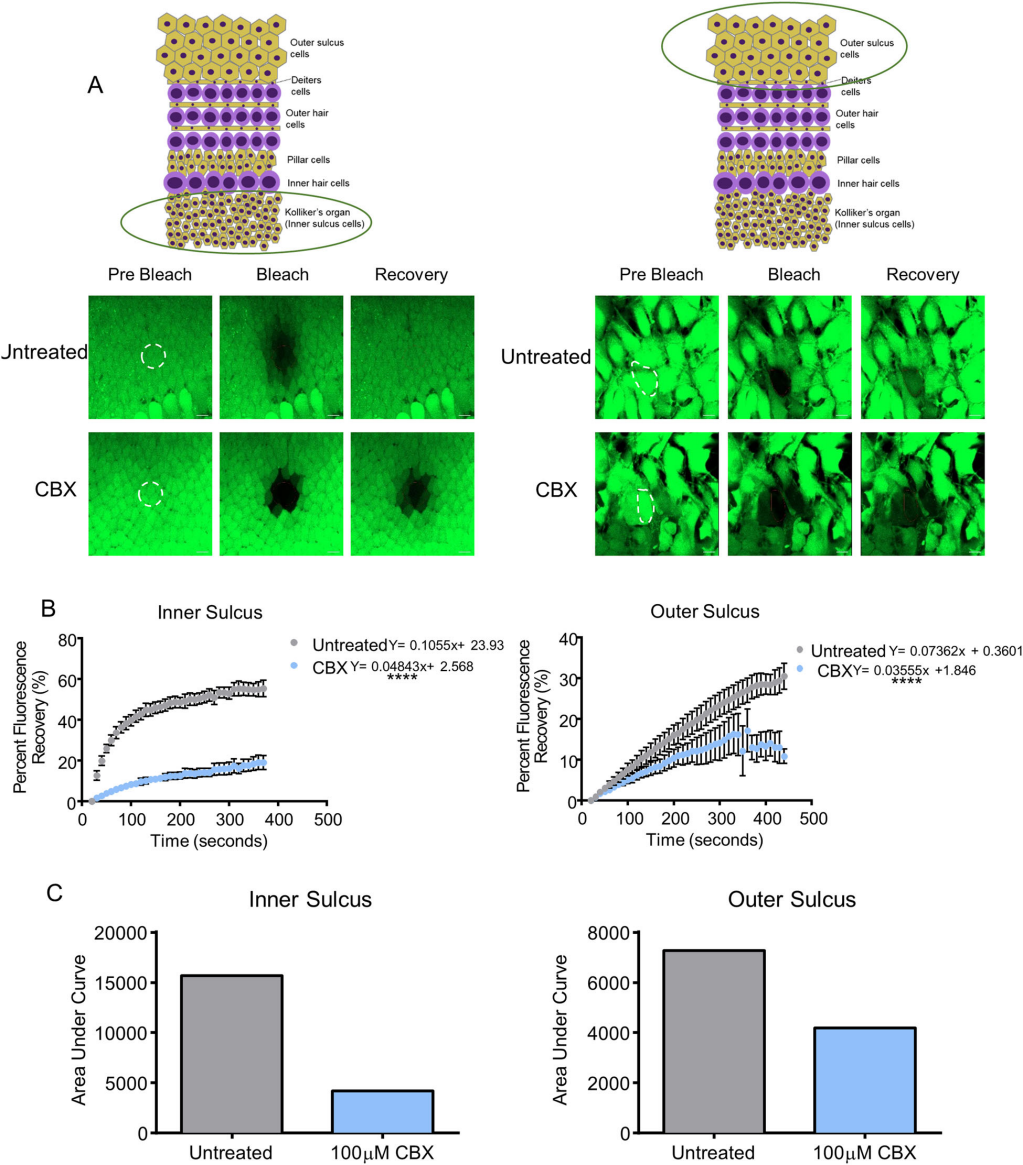
Carbenoxolone blocks calcein dye transfer in cochlear supporting cells. **a** Representative images of prebleach, bleached, and dye recovery after photobleach of calcein fluorescence in untreated or CBX-treated cochlear culture regions. Dashed areas represent photobleached regions. The photobleached region of inner sulcus cells was 288.5 µm^2^. **b** Percentage of fluorescence recovery compared with baseline in the inner and outer sulcus regions of untreated or 100 μM CBX-treated cochlear cultures. **c** Area under the curve for both inner and outer sulcus regions revealed that CBX was an effective inhibitor of dye transfer. *N* = 3, *n* = 6 in each group and for each region. CBX carbenoxolone treated. Scale bars = 10 μm. Linear regression analysis of slopes performed for each region and area, ****p < 0.0001.

We next took a pharmacological approach to determine if blocking all cochlear gap junction function would attenuate the effect of cisplatin-induced cell death. To confirm that carbenoxolone (CBX) blocked GJIC in WT organotypic cochlear cultures, fluorescence recovery after photobleaching (FRAP) was performed. As expected, supporting cells in the Kolliker’s organ region were well coupled via gap junctions as WT cultures recovered ~60% of their initial fluorescence intensity after photobleaching (Fig. 7a, b). However, CBX-treated cultures exhibited reduced fluorescence recovery (Fig.7a, b). Cells from untreated cultures in the outer sulcus region of cochlear cultures were also well coupled, but to a lesser degree than cells of Kolliker’s organ region, as they recovered ~30% of their original fluorescence intensity (Fig. 7a, b). A linear regression analysis of the slopes of the lines and assessment of the area under the curve of the linear regression all confirmed that CBX treatment significantly inhibited GJIC in both inner and outer sulcus regions (Fig. 7a–c).

To assess whether CBX treatment altered Cx26 and Cx30 levels, organotypic cultures were treated with 100 μM CBX, 20 μM cisplatin, or a combination of 100 μM CBX + 20 μM cisplatin. At low magnification of cultures treated with CBX, limited immunolabeled Cx26 or Cx30 was detected as compared with the abundant signal found in untreated and cisplatin-treated cultures (Supplementary Fig. S3a). Similarly, at higher magnification, supporting cells of the inner sulcus and outer sulcus regions expressed abundant amounts of Cx26 and Cx30 in both the untreated and cisplatin-treated groups, but both isoforms were undetectable after CBX treatment (Supplementary Fig. S3b).

### Blocking gap junctions does not alter cisplatin-induced hair cell death

**Fig. 8:**
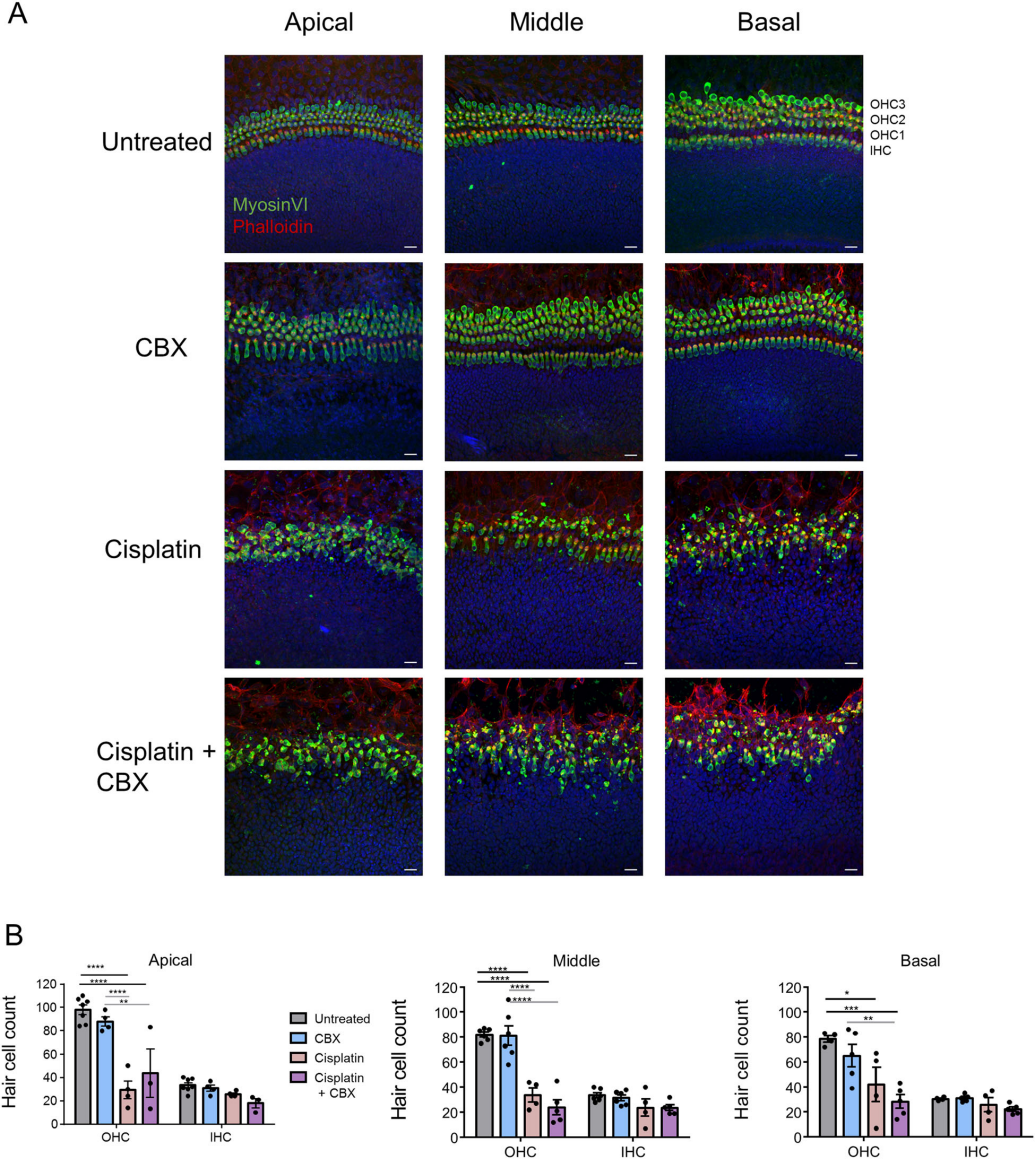
Blocking gap junctions does not impact cisplatin-induced hair cell loss. **a** Representative confocal images of hair cells in WT cultures that were untreated or treated with 100 μM carbenoxolone (CBX), 20 μM cisplatin, or 100 μM CBX and 20 μM cisplatin (cisplatin + CBX) for 48 h. MyosinVI is denoted in green, phalloidin is in red, and nuclei are in blue. **b** Quantification of hair cell counts from all four treatment groups at the apical, middle, and basal regions. *N* values; untreated = 4–7, CBX = 4–6, Cis = 4, Cis+ CBX = 3–5. OHC outer hair cell, IHC inner hair cell. Bars represent mean ± standard error. Scale bars = 20 μm. Two-way ANOVA’s and subsequent Tukey’s post hoc tests were performed for each region. *p < 0.05, **p < 0.01, ***p < 0.001, ****p < 0.0001.

Since CBX was a found to be an excellent blocker of cochlear connexins we assessed if this caused a change in hair cell susceptibility to cisplatin-induced cell death. Representative images of hair cells revealed that CBX treatment did not induce cell death, but cisplatin alone or in combination with CBX was a potent inducer of hair cell death (Fig. 8a). Specifically, hair cell counts revealed that when 20 μM cisplatin and 100 μM CBX were co-administered, there were no significant differences in hair cell number compared with cisplatin treatment alone (Fig. 8b). Thus, blocking gap junctions in cochlear organotypic cultures does not affect cisplatin-induced hair cell loss.

### WT and Cx43 KO HEI-OC1 cells undergo different mechanistic routes leading to cisplatin-induced cell death

To examine the mechanisms of action of cisplatin-induced cell death in gap junction competent and deficient HEI-OC1 cells, reporter proteins for intrinsic mitochondrial cell death and endoplasmic reticulum (ER) stress were assessed. First, to assess whether WT or Cx43 KO cells have differential susceptibility to reactive oxygen species (ROS) accumulation, mRNA expression of antioxidant enzymes were examined. Forty-eight hours of 15 μM and/or 30 μM cisplatin treatment triggered an increase in the antioxidants manganese superoxide (MnSOD), catalase, and glutathione peroxidase (GPx1) in Cx43 KO, but not in WT cells (Supplementary Fig. S4a). Pro-apoptotic Bax and anti-apoptotis Bcl-2 mRNA ratio was elevated in both cell lines, indicating a role for the mitochondria in this process. However, WT HEI-OC1 cells exhibited a more profound upregulation compared with Cx43 KO cells, suggesting enhanced mitochondrial dysfunction. In addition, western blots showed that Bax protein expression in WT HEI-OC1 cells was significantly increased after treatment with 15 μM cisplatin, however no differences were found in Cx43 KO cells (Supplementary Fig. S4b, c).

To evaluate if ER stress is at the root of cisplatin-induced cell death, the expression of immunoglobulin protein (BiP), an ER chaperone protein upregulated during ER stress, was examined. Immunofluorescence revealed that BiP was readily detected after cisplatin treatment in both WT and Cx43 KO HEI-OC1 cells, and was localized to the ER compartment as evident by co-localization with protein disulfide isomerase (PDI) (Supplementary Fig. S5A). Western blots revealed that BiP was found in higher abundance in Cx43 KO but not WT cells after cisplatin treatment compared with untreated cells (Supplementary Fig. S5b, c).

## Discussion

The reason(s) why the inner ear is so highly susceptible to cisplatin damage is not entirely clear. Gap junctions are highly enriched within the organ of Corti and may mediate (potentially toxic) small molecule exchange between connecting cells. Related studies have shown that Cx43-mediated GJIC plays a bystander role in cancer cell lines where downregulating Cx43 leads to protection against cisplatin-induced cell death^44–46^. Although the role of Cx43 in hearing and its localization pattern in the cochlea remain controversial, we have shown for the first time that Cx43 is expressed at low levels in organotypic cochlear cultures of neonatal mice. Interestingly, both Cx26 and Cx30 expression were more pronounced in the apical (low-frequency) region of organotypic cochlear cultures compared with the basal (high-frequency) region, a finding that has also been reported in guinea pigs^47^. This is of particular interest due to the fact that hearing loss generally occurs primarily in the high-frequency areas^48,49^, a phenomenon that could be due to lowered Cx26 and Cx30 expression within this region.

Although Cx43 has been reported to exacerbate cisplatin-induced cell death, these studies have primarily been examined in cancer cell lines, including testicular^34^, lung^44,50^, ovarian^44^, and breast^51^. Recently, a study showed that although Cx43 increased cell death in cisplatin-treated testicular cancer cells, this effect was reversed in noncancerous testicular cells where Cx43 downregulation led to enhanced cisplatin-induced cell death^34^. This suggests that Cx43 may play distinct roles with either propagation of death signals (bystander) or beneficial signals (Good Samaritan) in cisplatin treatment, depending on the cell status. To further examine the role of Cx43 in cisplatin-induced ototoxicity, Cx43^I130T/+^ and Cx43^G60S/+^ mutant mice harboring a moderate and severe reduction of Cx43-mediated GJIC, respectively, were used. In the case of Cx43^I130T/+^ mutant mice, we have established that the expected level of Cx43-mediated GJIC is ~50% while Cx43^G60S/+^ mutant mice have only ~20% functional Cx43^37,38^. We know that Cx43^G60S/+^ mice exhibit severe hearing loss, thereby linking Cx43 to hearing function^23^. Organotypic cochlear cultures from these mutant mice provide an opportunity to assess Cx43 function on cisplatin-induced hair cell death in vitro. Clinically, the plasma concentration range of cisplatin achieved during most cancer treatments is approximately 1.7 µM–25 µM^39,40^, thus concentrations used in our study are comparable with the clinical setting. We found that although cisplatin treatment caused significant outer hair cell loss compared with untreated cultures, neither mutant mouse showed any differences in hair cell loss compared with WT littermates. Interestingly, cultures from Cx43^G60S/+^ mutant mice had a significantly increased number of CC3-positive hair cells, suggesting that these hair cells may be undergoing enhanced apoptosis possibly at a later time point due to the reduced level of functional Cx43^37,38^. Cx43 KO HEI-OC1 cells had significantly higher cell viability compared with WT cells only when treated with 10 µM cisplatin for 24 h, which is consistent with another report where the levels of Cx43 in HEI-OC1 cells were reduced using siRNA^35^. However, higher cisplatin concentrations eliminated this protection, suggesting that cisplatin toxicity caused cell death irrespective of GJIC. Further supporting the notion that GJIC plays a minimal role in cisplatin-induced cell death, we found that pharmacological blockage of all gap junction channels with CBX had no effect on either resistance or sensitivity of organotypic cultured hair cells to cell death.

Connexins are expressed in cochlear supporting cells and not in hair cells in vivo^52–54^, thus blocking gap junctions may not have a direct effect on cisplatin entry and/or propagation in hair cells themselves. Hair cells express many uptake transporters for cisplatin such as Ctr1, OCT1, and OCT2^55–57^, allowing cisplatin to directly enter hair cells. Two copper transporting ATPases, ATP7A and ATP7B, mediate cisplatin efflux from cells and are expressed in pillar cells and hair cells, respectively^57^. Although hair cells are suggested to be the primary target of cisplatin, damage to supporting cells may also play a role in cisplatin-induced cell death through indirect mechanisms. A previous study showed that after in vivo administration of cisplatin in rats, supporting cell morphology was altered prior to hair cell loss^58^. This is in line with our findings that altered architecture of the supporting cells may play a key role. It has been well documented that when the cochlea is injured, nearby supporting cells expand, extrude, and eventually phagocytose the injured hair cells^59–61^. Cisplatin also disrupts the actin cytoskeleton in supporting cells where they form a “glial scar” to maintain the proper integrity of the sensory epithelium^62^. Taken together, these data suggest that although hair cell loss has been mostly documented as a consequence of cisplatin treatment, supporting cells in proximity to hair cells have altered morphology that may impact hair cell death. In addition, architectural changes in supporting cells occur independent of Cx43-based GJIC, suggesting the toxic or beneficial signals do not pass through the gap junction-rich supporting cells.

In the cochlea, Cx26 and Cx30 have been shown to form heteromeric and/or heterotypic gap junction channels in most cochlear supporting cells^33,63,64^. Our study showed that cisplatin caused a reorganization of Cx26 and Cx30 in all organotypic cochlear cultures, reducing the number of hybrid gap junction channels. This rearrangement may give rise to distinct changes in the metabolome that can spread among the supporting cells, including propagation of cell death related toxic signals^65^. For example, a previous study showed that heteromeric Cx26/Cx30 cochlear channels had faster Ca^2+^ wave propagation than their homomeric counterparts^64^. Thus, we suspect that cisplatin alters the biochemical coupling status of cochlear supporting cells through the production of fewer heteromeric channels.

Carbenoxolone (CBX) has been widely used as a nonspecific gap junction blocker in many different tissues and cell lines including resident cells of the inner ear^41,42,66–68^. However, the mechanism of action remains unclear. In rat liver epithelial cells, CBX was shown to dephosphorylate Cx43, leading to a decrease in GJIC^69^. This study showed that organotypic cochlear cultures treated with CBX had significantly decreased GJIC and Cx26 and Cx30 protein expression, suggesting a novel mechanism of action for CBX in the inner ear. In support of our findings that connexin levels are reduced after CBX treatment, previous studies showed that CBX caused a decrease in Cx43 levels in the prefrontal cortex of rats^70^, human glioma cells^71^, and human cerebral endothelial cells^72^. In contrast, bovine aortic endothelial cells (BAECs) treated with CBX had elevated levels of Cx43 mRNA and protein^73^. To our knowledge, our study is the first to show that CBX attenuates the protein levels of Cx26 and Cx30, suggesting a mode of action that may extend beyond blocking the function of gap junction channels.

Cisplatin causes DNA damage leading to activation of various cell death pathways, including the intrinsic mitochondrial pathway via the permeabilization of the mitochondrial outer membrane and subsequent caspase activation^74^. This in turn generates ROS while depleting antioxidants, leaving cochlear cells unable to rapidly excrete toxins due to its anatomically closed structure^75^. The ER stress pathway whereby a buildup of misfolded proteins causes cellular dysfunction has also been shown to be involved in cisplatin-induced cell death^76^. To examine these pathways, we evaluated the levels of various antioxidants, Bax (pro-apoptotic protein), and BiP (ER chaperone binding protein) as markers of mitochondrial apoptosis and ER stress, respectively. We showed that both WT and Cx43 KO cells underwent signature changes of the intrinsic mitochondrial pathway upon cisplatin treatment. Interestingly, Cx43 KO cells had increased levels of antioxidant mRNA expression compared with WT, possibly making them more resistant to ROS. In general, WT HEI-OC1 cells had significantly increased Bax protein expression (i.e., mitochondrial), whereas Cx43 KO HEI-OC1 cells had significantly increased BiP protein expression (i.e., ER) after cisplatin treatment. It is important to note that the cytoplasmic tail of Cx43 has been shown to bind Bax^77^, and thus, WT HEI-OC1 cells may undergo enhanced mitochondrial dysfunction because they have the capacity to bind to Bax. Taken together, these data suggest that WT cells had enhanced mitochondria dysfunction while Cx43 KO cells favored an ER stress response pathway.

In summary, this study sought to examine the role of GJIC in cisplatin-induced ototoxicity in two different cochlear-relevant models. Collectively, we found that GJIC did not influence the amount of cisplatin-induced hair cell death but did cause alterations in cochlear supporting cell morphology and reorganization of Cx26 and Cx30 gap junctions. This is the first study to show that HEI-OC1 cells were highly sensitive to cisplatin-induced cell death regardless of the presence of Cx43, although the absence of Cx43 leads to more cisplatin-induced ER stress. Future studies examining the roles of GJIC in cisplatin-induced ototoxicity in the in vivo inner ear should be evaluated to further understand the molecular processes involved. Overall, our studies are consistent with the notion that patients that harbor anyone of several dozen connexin gene mutations are not at increased risk of cisplatin-linked hair cell loss and deafness than patients lacking these mutations.

## Supplementary information

Supplementary Figure Legends

Supplementary Figure 1

Supplementary Figure 2

Supplementary Figure 3

Supplementary Figure 4

Supplementary Figure 5
