# Supplementary material for: Cisplatin-induced ototoxicity in organotypic cochlear cultures occurs independent of gap junctional intercellular communication
Source: Cell Death Dis. 2020 May 11;11(5):342. doi: 10.1038/s41419-020-2551-8 (PMC7214471; doi:10.1038/s41419-020-2551-8)
Supplement: Supplementary file 1 — Supplementary Figure Legends [file 41419_2020_2551_MOESM1_ESM.docx]

**Supplemental Figure 1. Cleaved caspase 3 is upregulated after cisplatin treatment.**

(A) Representative confocal images of organotypic cochlear cultures labelled for cleaved caspase 3 (CC3) both in untreated and in 20μM cisplatin treated cultures from Cx43^I130T/+^ mutant mice and WT littermates. CC3 is denoted in red, phalloidin is in green, and nuclei are in blue. (B) CC3-positive hair cells were quantified as hair cells labelled positive for CC3 and phalloidin. N values; WT untreated= 4-5, WT cisplatin= 5-7, Cx43^I130T/+^ untreated= 4, Cx43^I130T/+^ cisplatin= 4. Means represent mean ± standard error. Scale bars= 20μm. One-way ANOVA’s were performed for each cochlear region and subsequently a post hoc Tukey’s test was performed. *p<0.5, **p<0.01, ***p<0.001.

**Supplemental Figure 2. Assessment of Cx26 and Cx30 levels in organotypic cochlear cultures after cisplatin treatment.**

Double-labeled immunofluorescence for Cx26 and Cx30 was performed in WT and Cx43^G60S/+^ mutant mice before and after cisplatin treatment. Particle analysis plugin on ImageJ was used to quantify the expression and number of gap junctions in the inner sulcus region of littermate mice. (A) Cx30 and Cx26 average intensity levels were analyzed in Cx43^G60S/+^ mutant mice and their WT littermates in control cultures and after 20μM cisplatin treatment for 48 hours. (B) The number of Cx30 and Cx26 gap junction plaques were quantified. (C) Average gap junction plaque size was quantified. N values; WT untreated= 5, WT cisplatin= 4, Cx43^G60S/+^ untreated=6-7, Cx43^G60S/+^ cisplatin= 3-4. Means represent mean ± standard error.

**Supplemental Figure 3. Carbenoxolone reduced the number of Cx26 and Cx30 gap junctions in cochlear supporting cells.**

(A) Double-immunofluorescent labeling for Cx26 and Cx30 was performed on the basal region of organotypic cochlear cultures before and after carbenoxolone (CBX) and/or cisplatin treatment. (B) Higher magnification images of Cx30 and Cx26 in supporting cells of the inner sulcus region and (C) outer sulcus region. Cx30 is denoted in green, Cx26 is in red, phalloidin is in white, and nuclei are in blue. Scale bars= 20μm.

**Supplemental Figure 4. Cx43 KO HEI-OC1 cells have increased mRNA expression of antioxidant enzymes after cisplatin treatment.**

(A) Quantitative real time polymerase chain reaction (qRT-PCR) of antioxidant enzymes: manganese superoxide dismutase (MnSOD), catalase, and glutathione peroxidase (GPx1) from WT and Cx43 KO HEI-OC1 cells after cisplatin treatment. The ratio of pro-apoptotic Bax to anti-apoptotic Bcl-2 mRNA levels was calculated. Bars represent mean ± standard error from four independent experiments comprised of two independent Cx43 KO clones. N values; WT: N=4, Cx43 KO: N=7. (B, C) Western blot and quantification of Bax protein expression. GAPDH was used as a protein loading control. N values; WT: N=4, Cx43KO: N=5-7. Two-way ANOVA with Tukey’s post hoc test, *p<0.05, **p<0.01, ***p<0.001. #p<0.05 represents one-way ANOVA and subsequent Tukey’s post hoc test performed.

**Supplemental Figure 5. HEI-OC1 cells lacking Cx43 preferentially undergo an ER stress response after cisplatin treatment.**

(A) Representative confocal images of BiP expression and localization in WT and Cx43KO HEI-OC1 cells treated with saline, 15μM, or 30μM cisplatin for 48 hours. BiP is in green, PDI (an ER resident marker) is in red, and blue are nuclei. WT HEI-OC1 cells treated with thapsigargin, a known activator of ER stress were used as a positive control. (B) Western blot for BiP protein expression in saline and cisplatin-treated cells. GAPDH was used as a loading control. (C) Quantification of BiP protein expression in cells treated with saline (untreated), 15μM, or 30μM cisplatin for 48 hours. Bars represent mean ± standard error from four independent experiments comprised of two independent Cx43 KO clones. N values; WT: N=3-4, Cx43KO: N=5-8. Scale bars=10μm. Two-way ANOVA with Tukey’s post hoc test, *p<0.05. #p<0.05, ##p<0.01 represents one way-ANOVA followed by a Tukey’s post hoc test.
