# Supplementary figures and images for: Cisplatin-induced ototoxicity in organotypic cochlear cultures occurs independent of gap junctional intercellular communication
Source: Cell Death Dis. 2020 May 11;11(5):342. doi: 10.1038/s41419-020-2551-8 (PMC7214471; doi:10.1038/s41419-020-2551-8)

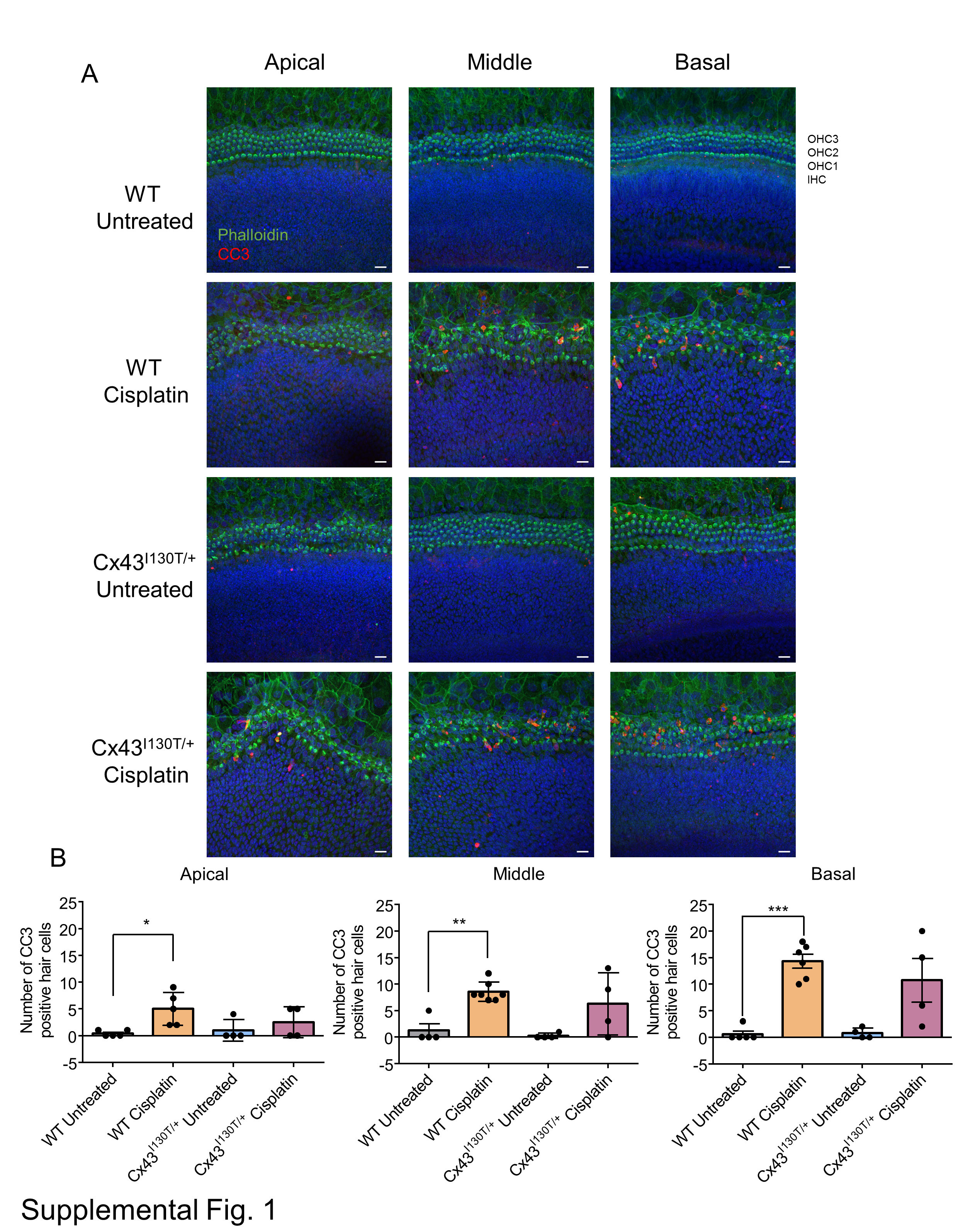

Supplement: Supplementary file 2 — Supplementary Figure 1 [file 41419_2020_2551_MOESM2_ESM.tif]

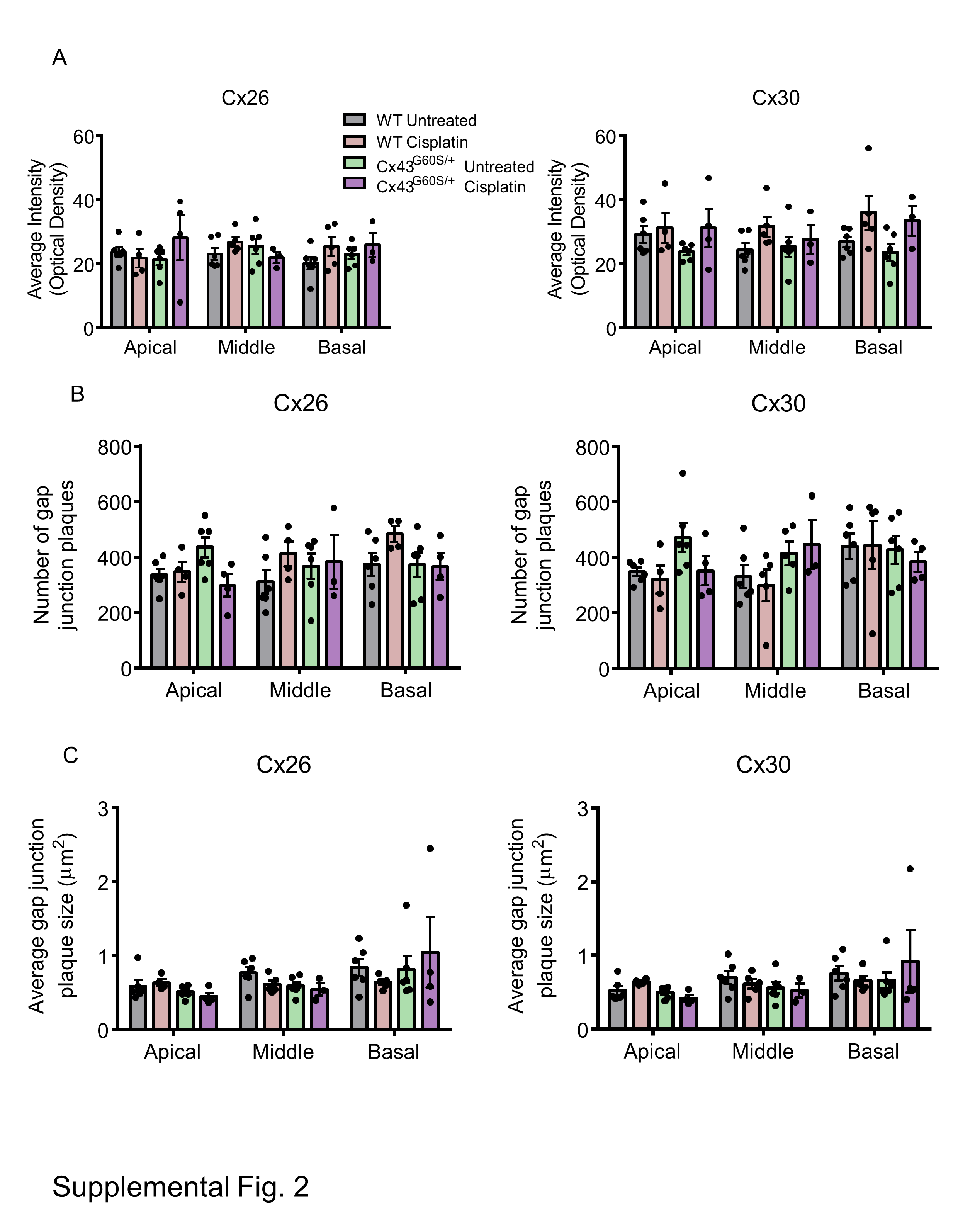

Supplement: Supplementary file 3 — Supplementary Figure 2 [file 41419_2020_2551_MOESM3_ESM.tif]

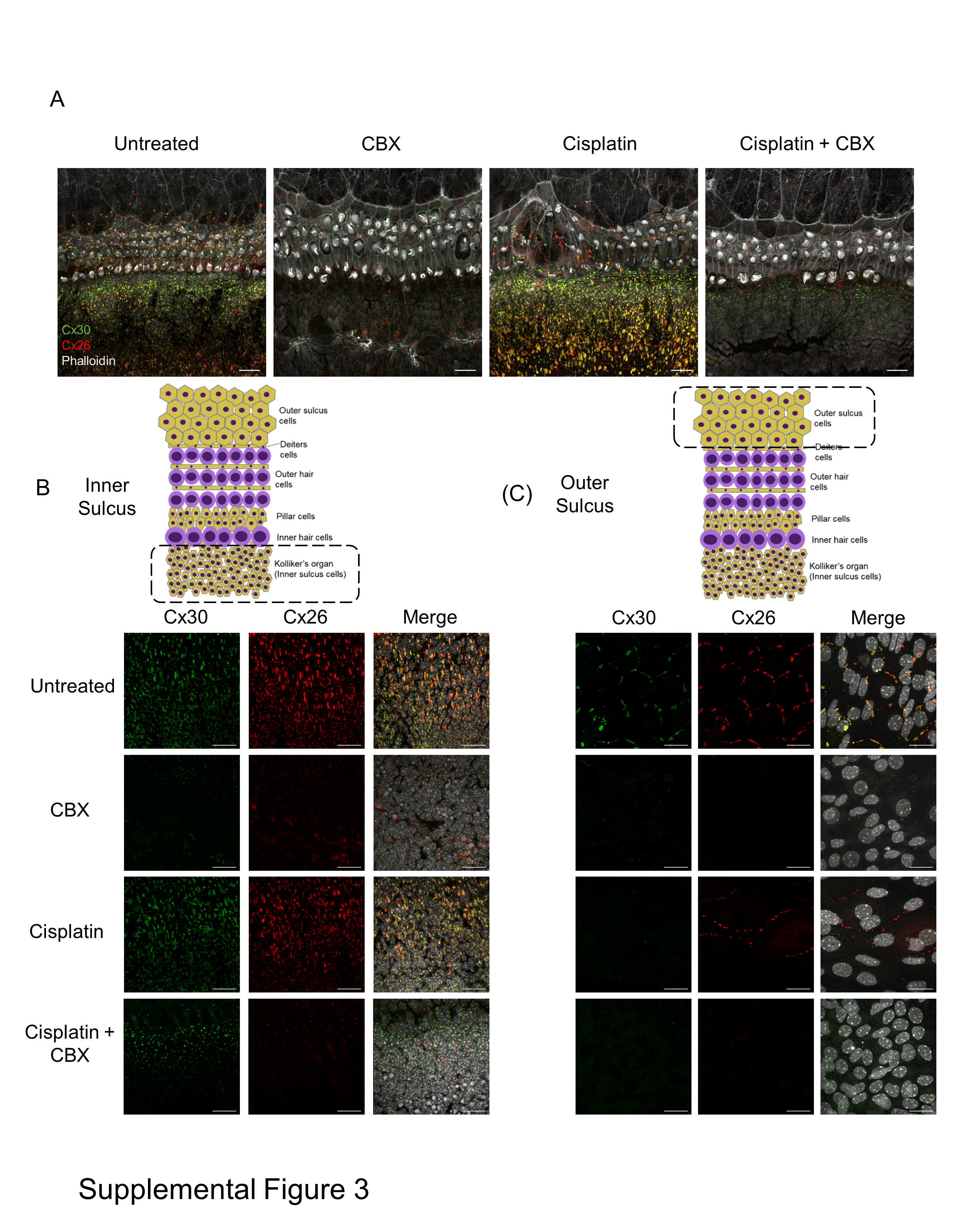

Supplement: Supplementary file 4 — Supplementary Figure 3 [file 41419_2020_2551_MOESM4_ESM.tif]

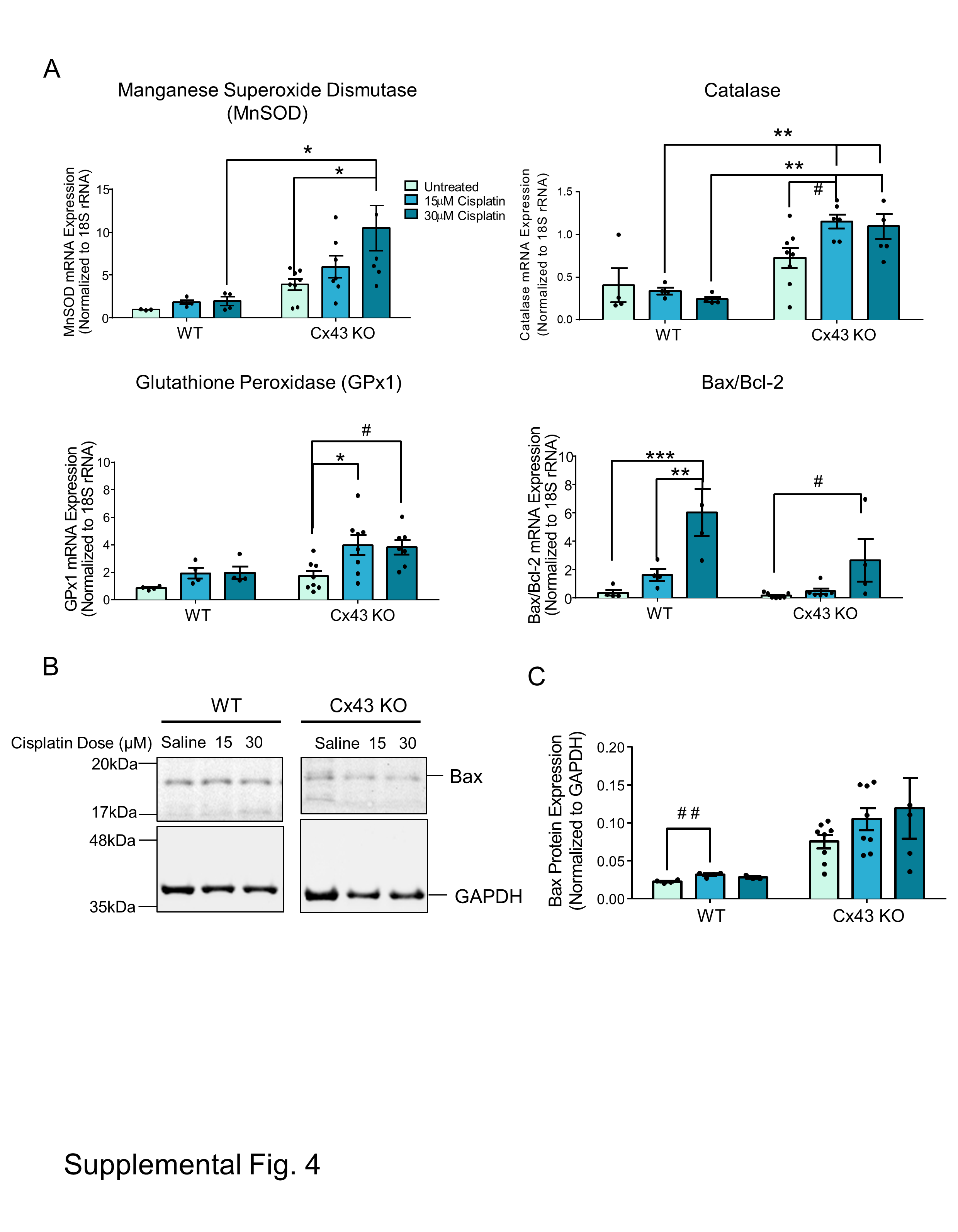

Supplement: Supplementary file 5 — Supplementary Figure 4 [file 41419_2020_2551_MOESM5_ESM.tif]

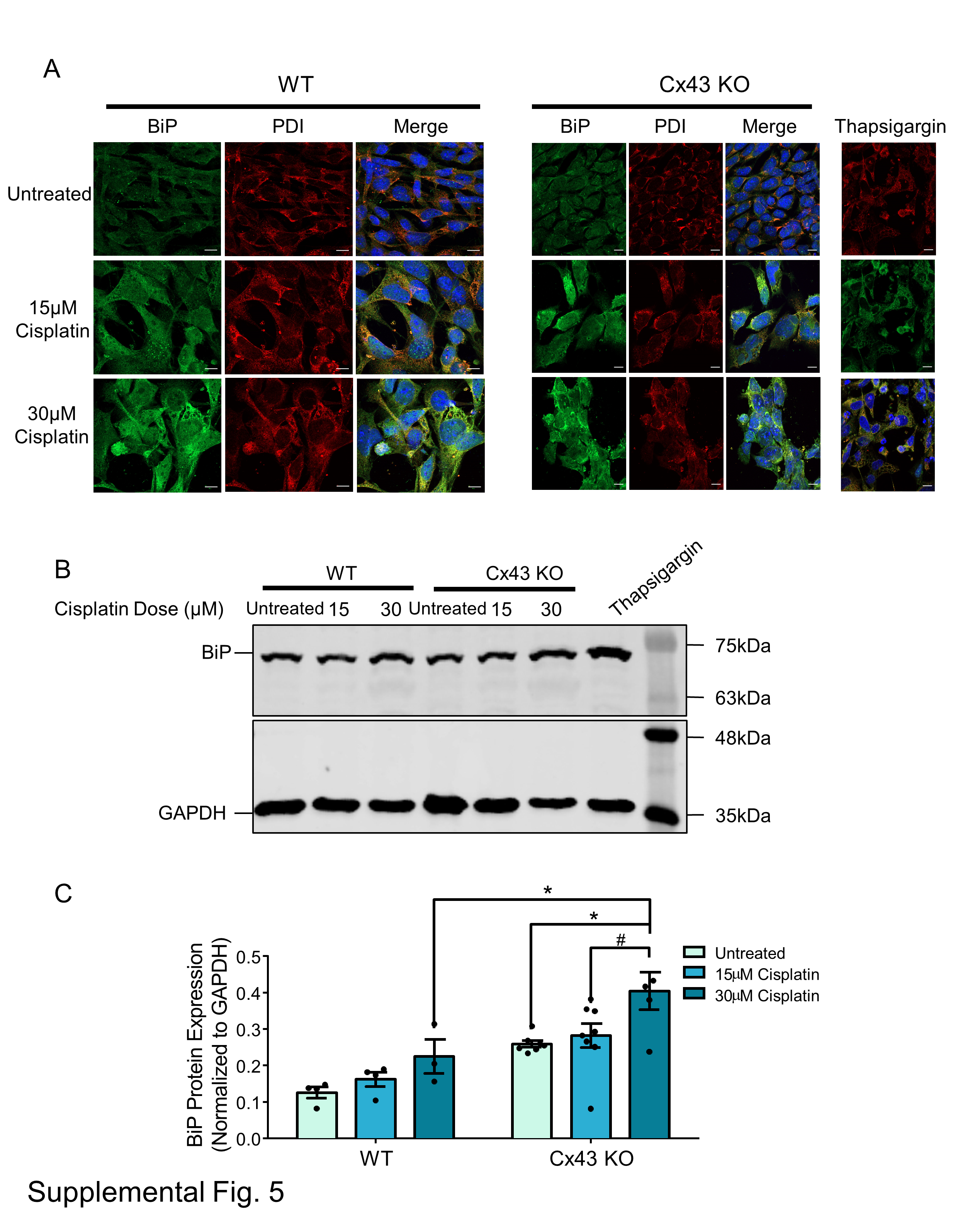

Supplement: Supplementary file 6 — Supplementary Figure 5 [file 41419_2020_2551_MOESM6_ESM.tif]
